# Lung cancer awareness and palliative care interventions implemented in low-and middle-income countries: a scoping review

**DOI:** 10.1186/s12889-020-09561-0

**Published:** 2020-09-29

**Authors:** Ugochinyere I. Nwagbara, Themba G. Ginindza, Khumbulani W. Hlongwana

**Affiliations:** Discipline of Public Health Medicine, School of Nursing and Public Health, University of KwaZulu-Natal, Durban, 4041 South Africa

**Keywords:** Lung cancer, Awareness, Palliative care, Interventions, Low-and middle-income countries

## Abstract

**Background:**

Lung cancer is the most diagnosed cancer worldwide. In low- and middle-income countries (LMICs), lung cancer is often diagnosed at a late stage due to poor knowledge and awareness of its signs and symptoms. Increasing lung cancer awareness is likely to reduce the diagnosis and treatment delays. The implementation of early palliative care has also been reported to improve a patient’s quality of life, and even survival. The aim of this scoping review was to map evidence on lung cancer awareness and palliative care interventions implemented in sub-Saharan Africa (SSA) and other LMICs.

**Methods:**

This scoping review was guided by Arksey and O’Malley’s framework. Databases such as the EBSCOhost, PubMed, Science Direct, Google Scholar, World Health Organization (WHO) library and grey literature were used to perform systematic searches of relevant articles. The methodological quality assessment of included primary studies was assessed using the Mixed Method Appraisal Tool (MMAT). NVivo version 10 software was used to perform the thematic content analysis of the included studies.

**Results:**

A total number of screened articles was 2886, with 236 meeting the eligibility criteria and 167 further excluded following abstract screening. Sixty-nine (69) articles qualified for full-article screening and 9 were selected for detailed data extraction and methodological quality assessment. Of the included nine studies, eight described at least one lung cancer warning signs and symptoms, while one described the effectiveness of palliative care for lung cancer. Eight articles recognized the level of lung cancer knowledge, risk factors awareness of warning signs and symptoms in LMICs, mostly Africa and Asia.

**Conclusions:**

Most of the participants were aware of tobacco use as the major risk factor for lung cancer but lacked knowledge on the other pre-disposing risk factors. Evidence on palliative care is scarce, therefore, awareness interventions packaged with evidence on the value of timely access to palliative care services in improving the quality of life of the lung cancer patients and their families, are required.

## Background

Globally, cancer is the second leading cause of death, and estimated to be responsible for 18.1 million cases and 9.6 million deaths in 2018 [1–3]. Lung cancer is the most commonly diagnosed cancer worldwide and the leading cause of cancer-related deaths, with approximately 2.1 million new lung cancer cases and 1.8 million deaths reported in 2018 [1]. The 2018 report by the World Health Organization (WHO) indicated that lung cancer was responsible for nearly one in five (18.4%) cancer-related deaths across the globe [1]. Increasing cancer-related mortality in low-and middle-income countries (LMICs), including sub-Saharan Africa (SSA), are attributable to aging and pervasive risk factors, including cigarette smoking, alcohol use, unhealthy diet and lack of physical activity [4–7].

In 2012, 65% of all cancer-related deaths worldwide, occurred in LMICs, with further increase likely to reach 75% by 2030 [3, 8], unless the situation is averted. In spite of a relatively lower incidence of cancer in LMICs, compared to their high-income countries (HICs) counterparts, cancer-related mortality is proportionally higher in LMICs, particularly in people younger than 65 years of age [3].

In LMICs, including SSA, lung cancer is often diagnosed at an advanced stage, which has been the main cause of treatment delays [7, 9–12], at times, leading to the disease advancing to terminal stages [13, 14]. Therefore, increasing awareness and early recognition of signs and symptoms of lung cancer at community level, is paramount to the reduction of cancer morbidity and mortality in LMICs [5].

About 70% of lung cancer-related deaths, worldwide, are associated with tobacco use, with smokers being twenty times more likely to die from lung cancer-related conditions than their non-smoking counterparts [15, 16]. The prevalence of smoking in LMICs is on the rise, due to, among other things, the affordability of tobacco products, and this increase has been predicted to continue, unless appropriate stringent tobacco control interventions are implemented [17]. Cancer can be prevented by avoiding risk factors and implementing prevention strategies like smoking cessation and tobacco control which are viewed as the primary prevention of lung cancer [18]. However, for those who are already living with the disease, palliative care may be a viable option, which needs to be incorporated into the care plan.

The WHO defines palliative care as “an approach that improves the quality of life of patients and their families facing the problems associated with life-threatening illness, through the prevention and relief of suffering by means of early identification and impeccable assessment and treatment of pain and other problems, physical, psychosocial, and spiritual” [19]. Palliative care focuses on providing relief from the symptoms and stress related to life-threatening illness, including lung cancer, while improving the quality of life for both the patient and the family members. Implementation of early palliative care has been proven to ease symptom burden, improve patient’s quality of life, and most importantly improve survival [20, 21]. The importance of palliative care cannot be overemphasised given the projections indicating that SSA countries will have more than 85% increase in cancer burden by 2030, with Morhason-Bello et al. [22] and Stefan et al. [23] proposing further interventions to include cancer awareness, research, advocacy, workforce capacitation, training, high quality care and funding investments [23], in order to avert this situation. Of concern, a systematic review by Austoker et al. [24] found limited evidence on the effectiveness of community-level interventions to promote cancer awareness. Patients with lung cancer are rarely identified early, with more than 90% of them being symptomatic at the time of diagnosis and experiencing, at least, two to three symptoms on average [25, 26]. Cough is the most common symptom, which is considered to be a good prognostic indicator of lung cancer [12, 13, 25].

Evidence on lung cancer awareness and palliative care interventions implemented in LMICs, including SSA, is rare. The findings of this scoping review will better our understanding of lung cancer awareness and palliative care interventions implemented in LMICs and identify knowledge gaps for further research.

## Methods

A scoping review was adopted for this study as the appropriate approach to map literature on available evidence the lung cancer awareness and palliative care interventions implemented in low-and middle-income countries, including SSA. This study was guided by the Arksey and O’Malley’s [27] methodological framework for scoping reviews. The framework stipulates the following steps: identification of the research question; identification of the relevant studies; study selection; charting the data; and collating, summarizing and reporting the results. A quality assessment of the included primary studies as recommended by Levac et al. [28] was also included in the study. The PRISMA (Preferred Report Items for Systematic and Meta-Analysis) [29] flow diagram was used for the selection and screening of the studies.

### Identification of the research question

Our research question was “what is known from the existing literature on the lung cancer awareness and palliative care interventions implemented in low-and middle-income countries, including SSA?”

### Identification of the relevant studies

In order to identify relevant studies addressing the research question, we performed a scoping review which included all study designs published in peer-reviewed journals and grey literature. Databases such as the EBSCOhost, PubMed, Science Direct, Google Scholar and World Health Organization (WHO) library were used to perform systematic searches of relevant articles. The following keywords such as ‘Lung cancer’, ‘Awareness’, ‘Palliative care’, and ‘Interventions’ were included during the search. Boolean terms such as ‘AND’ and ‘OR’ were used to separate the keywords during the search. Medical Subject Headings (Mesh) terms were also included in the search as included in Additional file 1. Our searches were confined to the literature published in English language from January 2008 to June 2018. These timelines were motivated by the initial searches of literature revealing that most relevant studies were conducted after 2008, in addition to a 10-year period being considered likely to yield a comprehensive literature in the area of research interest.

### Study selection

We screened the titles from the databases with guidance from the inclusion and exclusion criteria. All studies with relevant titles for this research were exported to an endnote library and duplicates were removed. Two reviewers (UIN and MO) conducted abstract and full article screening independently and were guided by the eligibility criteria. Discrepancies in reviewers’ responses at abstract and full article screenings were resolved through discussion and a third reviewer was consulted when the reviewers were unable to resolve their disagreements through discussion.

### Inclusion criteria

Included studies met the following criteria:

- Studies published in English language from January 2008 to June 2018.
- All study designs published in peer-reviewed journals and grey literature
- Articles on lung cancer awareness and/or palliative care interventions in adults.
- Studies on lung cancer awareness interventions implemented in LMICs and whose discussions and conclusions demonstrated generalizable and/or transferable findings to SSA settings.

### Exclusion criteria

The following studies were excluded:

- Studies not available in English language and published before January 2008.
- Studies on lung cancer awareness and palliative care interventions in children.
- Articles on lung cancer awareness interventions implemented in High-Income-Countries (HICs).

### Charting the data

NVivo version 10 was used to organize data extracted from each article into different themes. Information extracted from the selected studies were organized and categorized as follows: author and year, study setting, aim, study design, population, mean/age range of participants, percentage of male and females, level of knowledge about lung cancer, awareness of signs and symptoms, lung cancer awareness of risk factors and most relevant findings.

### Collating and summarising findings

The extracted evidence was repeatedly reviewed to improve the quality of collated and summarized findings. A thematic content analysis of the data extracted from the included studies was performed to identify additional contextual factors (e.g. knowledge about lung cancer, awareness of risk factors, signs and symptoms for lung cancer, and palliative care interventions).

### Quality of evidence

Quality assessment of included studies was performed using the Mixed Method Quality Appraisal Tool (MMAT) Version 2011 [30]. Two independent reviewers (UIN and MO) assessed the quality of the included studies, using the following domains: the appropriateness of the research question, data collection, data analysis, accuracy of sampling methodology, author’s acknowledgement of possible biases and conclusion. An overall percentage quality score for each of the included studies was calculated and interpreted as <50% (low quality), 51–75% (average quality) and 76–100% (high quality).

## Results

### Screening results

**Fig. 1:**
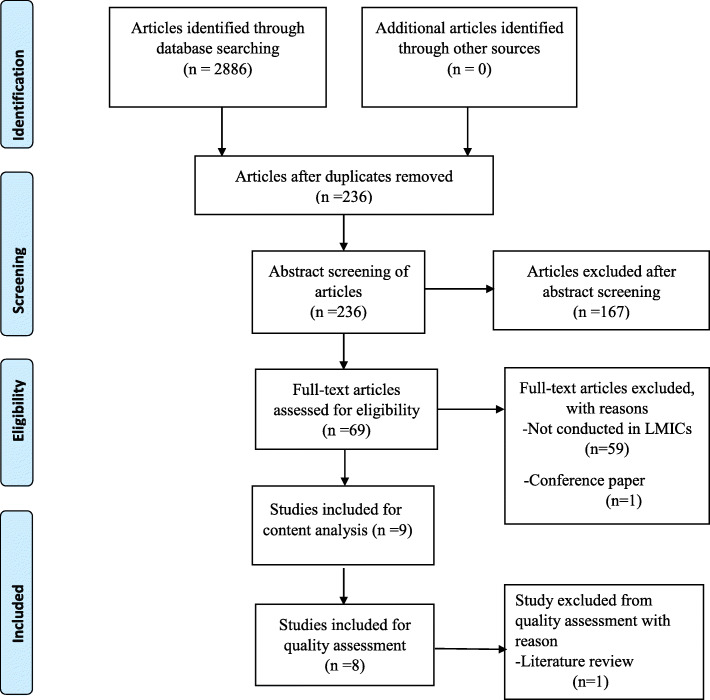
PRISMA flow diagram demonstrating the selection and screening of studies

A total of 2886 articles were identified after the database search **(**Fig. 1**)**. Following title screening and deletion of duplicates, 236 eligible studies were identified. A total of 167 articles were further excluded after abstract screening by two researchers, thereby reducing the articles eligible for full-article screening to 69 articles. Finally, 9 articles were selected for detailed data extraction, and subjected to methodological quality assessment. Degree of agreement between reviewers was 77.78% versus 80.25% expected by chance, and this constituted a considerably poor agreement between screeners (Kappa statistic = − 0. 13 and *p*-value > 0.05). Nevertheless, the McNemar’s chi-square statistic showed that there was not a statistically significant difference in the proportions of yes/no answers by reviewer with *p*-value > 0.05 **(**Additional file 2**).**

### Characteristics of included studies

All included studies were conducted in LMICs and published between 2008 and 2018, resulting in a total sample size of 3563 participants from primary studies. The majority of the participants were males in six studies [20, 31–35], two studies had a slight female preponderance [36, 37], while one study [21] was a literature review. Of nine studies included, 6 were cross-sectional studies [31–34, 36, 37], one was a pre-test and post-test study design [35], one was a prospective study [20] and the last one was a literature review [21]. Of the nine studies included in the review, eight showed at least one lung cancer warning signs and symptoms [20, 31–37], while one study described the effectiveness of palliative care for lung cancer [21]. The participants from one study had a good knowledge of lung cancer [37], one had a moderate knowledge [34], four studies had low level of knowledge [31, 33, 35, 36] and one study stated that knowledge of lung cancer varied by socio-demographic factors [32]. Table 1 illustrates the characteristics of the included studies.

**Table 1:** Characteristics of the included studies

Author & year	Country	Study aim	Study design	Population (n)	Mean/Age range of participants	Percentage of males	Percentage of females	Level of knowledge about lung cancer	Level of lung cancer awareness of signs and symptoms	Lung cancer awareness of risk factors	Relevant findings
**Al-Naggar, R. A. and 2013** [31]	Malaysia	To determine the lung cancer knowledge among male secondary school teachers	A cross-sectional study	150 male secondary school teachers	35.6 ± 6.5 (SD)	N/A	N/A	Low	Good	Majority were aware that the main risk factors for lung cancer was cigarette and second-hand smoking	The main preventive measures were smoking cessation, avoiding second-hand smoking and unnecessary chest x-ray
**Chawla, Rachit. et al and 2010** [32]	Nepal	To evaluate the awareness and assessment of risk factors for lung cancer	A cross- sectional study	240 subjects	33.4 ± SD 11.4 years	57.5%	42.5%	Varied significantly by socio-demographic factors	N/A	100% of males and 88% of females were aware that smoking was the primary risk factor for lung cancer	Knowledge of smoking as a risk factor for lung cancer varied by socio-demographic factors
**Desalu, O. O. and 2016** [33]	Nigeria	To determine the awareness of signs and risk factors for lung cancer and the anticipated delay before seeking medical care	A cross- sectional study	1125 adults	33 ± 10 years	51.4%	57.5%	Low	Low	More than half of the participants recognized that smoking, second-hand smoking and air pollution were risk factors for lung cancer	Awareness about lung cancer signs and risk factors were not satisfactory
**Loh, Jia Fui and 2018** [36]	Malaysia	To assess the knowledge of lung cancer among Malaysians and their willingness to undergo lung cancer screening	A cross- sectional study	385 responses	31.8 ± 12.8 years	44.7%	55.3%	Low	Low	Tobacco related factors and air pollution were the most known risk factors for lung cancer	There was a low awareness of the non-specific warning signs of lung cancer and majority of the participants were willing to undergo lung cancer screening
**Naskar, Subrata and 2017** [34]	West Bengal	To determine the level of lung cancer knowledge & awareness among secondary school teachers in West Bengal	A cross- sectional study	50 secondary school teachers	30–39 years	56.0%	44.0%	Moderate	Very low	Majority of the study participants mentioned that smoking was the most common cause of lung cancer	Gender factor had an impact on knowledge & level of awareness regarding lung cancer
**Shankar, Abhishek and 2016** [35]	India	To measure the level of lung cancer awareness among women and the impact of awareness programs in adopting safer practices	Pre-test and Post-test design	182 teachers	42.4 years (range: 28–59 years)	N/A	N/A	Low	Low	The teachers indicated that smoking, second-hand smoke, family history and exposure to asbestos were some of the most correct risk factors for lung cancer	There was a substantial increase in the level of lung cancer knowledge and the adoption of safe practices after awareness of lung cancer resulted to a significant change in smoking and alcohol habits
**Zainuddin, Norafiza and 2018** [37]	Malaysia	To evaluate the knowledge of lung cancer and perception on its screening among IIUM Kuantan students	A cross- sectional study	186 students	21 to 29 years	N/A	N/A	Good	Good	Most of IIUM Kuantan students answered correctly for common lung cancer risk factors such as smoking, air pollution, occupational exposure and passive smoking	Good knowledge of lung cancer resulted in positive perception of lung cancer screening
**Li, Huiqin and 2016** [21]	China	N/A	Literature review	N/A	N/A	N/A	N/A	N/A	N/A	N/A	palliative care was the recommended standard of care for patients with advanced NSCLC
**Bülbül, Y and 2017** [20]	Turkey	To investigate the lung cancer symptoms and evaluate the approaches to alleviate these symptoms.	Prospective study	1245 lung cancer patients	61.8 ± 9.4 years	88.7%	11.3%	N/A	N/A	N/A	Symptoms are more severe in patients at the advanced stage of lung cancer, and palliative care was insufficient for most patients

### Quality of evidence from included studies

Of the 9 included studies, 8 primary studies underwent methodological quality assessment (Additional file 3) using the Mixed Methods Appraisal Tool (MMAT)-Version 2011 [30], while 1 study was a literature review, hence it did not undergo quality assessment [21]. Four of the studies attained a high quality score of 76–100% [20, 32, 33, 36], and the remaining four studies had an average quality score of 51–75% [31, 34, 35, 37]. None of the eight included primary studies that underwent quality assessment had low quality score of <50%, thereby rendering the risk of bias in the overall evidence as minimal.

### Themes from included studies

#### Knowledge about lung cancer

Of the nine studies, participants from one study had a good knowledge of lung cancer [37], and another one had a moderate knowledge [34]. In another study, knowledge about lung cancer varied widely, mainly by socio-demographic factors [32], whereas in other four studies, low level of knowledge about lung cancer were revealed [31, 33, 35, 36]. In two studies, 70.7% [31] and 40% [34] of teachers mentioned that lung cancer can be spread from person to person, this was in contrast to another study that 86% of the participants recognized that lung cancer was not transmissible from one person to another [37]. A study by Zainuddin et al., conducted amongst Malaysian undergraduate students showed that less than half (43.5%) of the students knew that exercise could reduce the risk of acquiring lung cancer [37]. It has been suggested that physical activity reduces the risk of developing lung cancer and improves quality of life [37]. A Malaysian study conducted among students found a good knowledge of lung cancer, as they also knew that not only males were affected by lung cancer [37]. This was in contrast with a very poor knowledge of lung cancer demonstrated by more than half of the teachers in West Bengal, with participants also incorrectly stating that lung cancer only affects males [34].

#### Awareness of lung cancer risk factors, signs and symptoms

Out of the nine included studies, participants from seven studies showed a good knowledge for lung cancer risk factors [31–37]. Two studies reported a good knowledge of lung cancer signs and symptoms [31, 37], three studies showed poor knowledge [33, 35, 36], one had a very poor knowledge [34] and one study was not specific on the participant’s knowledge of the signs and symptoms of lung cancer [32]. In two studies, 92% [34] and 91.3% [31] of the participants demonstrated knowledge of cigarette smoking as the main risk factor for lung cancer. A study by Chawla et al. [32], stated that 100% of males were aware of smoking as the main risk factor for lung cancer. Participants in three studies showed low level of awareness of lung cancer warning signs [33, 35, 36]. In a study conducted by Naskar et al. [34] in West Bengal, 92% of the participants mentioned ionizing radiation, asbestos and other cancer-causing substances as risk factors for lung cancer [34]. Contrary to this, another study conducted in Malaysia by Al-Naggar et al. [31], reported that 51.3% of the study participants were not aware of asbestos, ionizing radiation and other cancer causing substances as lung cancer risk factors [31].

#### Lung cancer awareness interventions

Pre-test regarding knowledge, attitude and practice related to lung cancer was piloted among women in various Indian colleges before the start of a Pink Chain Campaign, through a questionnaire and subsequently followed by a post-test at 1 year and 6 months using the same questionnaire [35]. In between the pre-test and post-test, awareness programs comprising of an interactive section and lectures on preventive measures of lung cancer were conducted, with more emphasis on tobacco and smoking. The awareness campaign significantly increased the knowledge of lung cancer risk factors, and its signs and symptoms at 6 months and this continued after 1 year, resulting in changes in smoking and alcohol habits [35]. More than 60% of teachers mentioned that newspapers and magazines were the primary sources of information regarding lung cancer, while about 30% teachers were informed by their doctors about lung cancer [35]. The Pink Chain Campaign showed that access to relevant information and better means of communication was necessary to intensify public awareness on the dangers of cigarette smoking [35].

#### Palliative care interventions

One study mentioned radiotherapy, supportive care and chemotherapy as options of palliative therapies for lung cancer [21]. The use of non-narcotic analgesics alone, or combined with narcotic analgesics were the most common pain relief used by, at least 50% of all lung cancer patients, as revealed in a Turkish study conducted by Bulbul et al. [20] and 30.2% of the patients received palliative radiation therapy for bone metastasis [20]. Higher levels of depression and anxiety were reported in female patients than their male counterparts [20]. A literature review by Li and Li [21] showed palliative care for patients with advanced NSCLC, as the recommended standard of care [21]. A study showed that home use of oxygen, and use of bronchodilator were higher among lung cancer patients [20].

## Discussion

Mapping evidence on the lung cancer awareness and palliative care interventions implemented in LMICs, including SSA, is critical, in order to inform recognition of lung cancer risk factors, signs and symptoms. While this scoping review was designed to focus on the SSA countries, the dearth of literature on the lung cancer awareness and palliative care interventions implemented in SSA region, necessitated that we included LMICs in our study setting. The main goal was to include studies from LMICs, whose findings demonstrated potentials for transferability and/or generalizability to settings in SSA.

This scoping review identified 9 articles published between 2008 and 2018, eight of which recognized the level of lung cancer knowledge, risk factors and awareness of warning signs and symptoms in LMICs, mostly in Africa and Asia [20, 31–37]. Our findings demonstrated a gap in literature on individual and community level interventions promoting lung cancer awareness and palliative care in SSA specifically and LMICs generally. Most of the included primary studies were cross-sectional studies and did not mention interventions implemented despite cross-sectional designs being ranked lower in the hierarchy of evidence. The major symptoms of lung cancer as reported by the included studies were chest pain, coughing out blood, lack of appetite, pain, difficulty in breathing and tiredness [31, 33–37]. All the reviewed studies advocated for educating the public on how to recognize the signs and symptoms and risk factors of lung cancer, as the necessary intervention. Available evidence from our reviewed studies suggests that tobacco use is the most recognized risk factor for lung cancer, with majority of the participants believing that second-hand smoking and air-pollution were also important risk factors for lung cancer [31–37]. This may be indicative of the effectiveness of anti-smoking campaigns in flagging the harmful effects and dissuading the members of the public from the cigarette smoke [36]. There remains a poor recognition of the early signs of lung cancer in LMICs, and this calls for urgent awareness interventions directed at both the public and the health professionals alike [38]. Lung cancer preventive measures identified by our study were smoking cessation, avoidance of second-hand smoke and unnecessary chest x-rays, as well as a total ban of smoking in public places and institutions [31, 34]. A study stated that no less than 50% of all lung cancer patients used non-narcotic analgesics alone or combined it with narcotic analgesics for pain relief [20], and a large number of patients had unmet needs, in so far as lung cancer is concerned [20]. While most patients reported having continuous symptoms, a substantial number of patients with dyspnea and pain were not getting any treatment [20]. Early palliative care for lung cancer patients is therefore recommended for the relief of pain and other distressing symptoms while improving the quality of life for both the patients and their families. This study suggested that exercise may reduce the risk of getting lung cancer [37]. A study by Shankar et al. [35], during a Pink Chain Campaign found that the general awareness of signs and symptoms, screening modalities and risk factors of lung cancer improved after a year [35]. However, interventions such as that of the Pink Chain Campaign remain few and far in between. Therefore, it is necessary to increase the awareness of lung cancer signs and symptoms through the media and other relevant campaigns.

### Strengths and limitations

This study reaffirmed the value of scoping reviews in highlighting the evidence gaps in a given field. In this case, our scoping review revealed dearth of evidence on the lung cancer awareness and palliative care interventions in SSA specifically and LMICs in general. This study provides an opportunity for researchers to conduct empirical research to close the identified research gaps. The systematic approach followed in this study, using different databases and search strategies (electronic and manual), were the noteworthy strengths. However, despite these strengths, there is still a possibility that relevant articles were omitted, especially since our search was limited to studies published in English, from January 2008 to June 2018 in LMICs. It is possible that one or more good quality and relevant articles were published before January 2008, the period that fell outside the parameters for this study.

## Conclusions

This study highlighted the lung cancer awareness and palliative care interventions implemented in LMICs. Our study identified some evidence on interventions delivered to individuals during a Pink Chain Campaign, which showed that the general awareness of signs and symptoms and risk factors of lung cancer improved after 1 year alongside healthy practices linked to alcohol consumption and smoking. However, more LMICs, especially SSA, should emulate this campaign in their settings. While most of the participants were aware of tobacco use as a risk factor for lung cancer, majority still had limited knowledge on the other pre-disposing risk factors. Our study found limited evidence on palliative care, and majority of the patients continually suffered from symptoms and unmet needs. Therefore, there is an urgent need for the introduction of timely access to palliative care from diagnosis to end of life, in order to improve the quality of life for the lung cancer patients and their families. Health education activities against smoking should be implemented in schools, universities and the communities. Similarly, awareness programmes and campaigns should be conducted regularly, in order to increase lung cancer knowledge and warning signs.

## Supplementary information

**Additional file 1.** Search strategy.

**Additional file 2.** Degree of agreement calculation.

**Additional file 3.** Quality assessment of included studies.

## Data Availability

All data generated or analysed during this study are included in this published article and its supplementary information files.

## Abbreviations

GP: General practitioner
HICs: High- income countries
LMICs: Low- and middle-income countries
MeSH: Medical subject headings
MMAT: Mixed method appraisal tool
NSCLC: Non-small cell lung cancer
PRISMA: Preferred report items for systematic and meta-analysis
SSA: Sub-Saharan Africa
WHO: World health organization
