# Supplementary material for: Lung cancer awareness and palliative care interventions implemented in low-and middle-income countries: a scoping review
Source: BMC Public Health. 2020 Sep 29;20:1466. doi: 10.1186/s12889-020-09561-0 (PMC7526234; doi:10.1186/s12889-020-09561-0)
Supplement: Supplementary file 1 — Additional file 1. Search strategy. [file 12889_2020_9561_MOESM1_ESM.docx]

**Additional file 1: Appendix 1.** Search strategy.

(("lung neoplasms"[MeSH Terms] OR ("lung"[All Fields] AND "neoplasms"[All Fields]) OR "lung neoplasms"[All Fields] OR ("lung"[All Fields] AND "cancer"[All Fields]) OR "lung cancer"[All Fields]) AND ("awareness"[Subheading] OR "awareness"[All Fields] OR "awareness"[MeSH Terms])) AND ("interventions"[MeSH Terms] OR ("interventions"[All Fields]) AND ("2008/01/01"[PDat] : "2018/06/11"[PDat] AND "humans"[MeSH Terms]).

(("lung neoplasms"[MeSH Terms] OR ("lung"[All Fields] AND "neoplasms"[All Fields]) OR "lung neoplasms"[All Fields] OR ("lung"[All Fields] AND "cancer"[All Fields]) OR "lung cancer"[All Fields]) AND ("early palliative care"[Subheading] OR "early palliative care"[All Fields] OR "early"[MeSH Terms])) AND ("palliative care"[MeSH Terms] OR ("palliative"[All Fields] AND "care"[All Fields]) OR "palliative care"[All Fields]) AND ("2008/01/01"[PDat] : "2018/06/11"[PDat] AND "humans"[MeSH Terms]).
