# Supplementary material for: Lung cancer awareness and palliative care interventions implemented in low-and middle-income countries: a scoping review
Source: BMC Public Health. 2020 Sep 29;20:1466. doi: 10.1186/s12889-020-09561-0 (PMC7526234; doi:10.1186/s12889-020-09561-0)
Supplement: Supplementary file 2 — Additional file 2. Degree of agreement calculation. [file 12889_2020_9561_MOESM2_ESM.docx]

**LUNG CANCER AWARENESS AND PALLIATIVE CARE INTERVENTIONS IMPLEMENTED IN AFRICA: a systematic scoping review**

**Full Article Screening**

|  | **Author and Year** | **Reviewer 1: Response** | **Reviewer 2: Response** |
| --- | --- | --- | --- |
| **1** | Al-Naggar, R. A. et al and 2013 | 1 | 0 |
| **2** | Bülbül, Y. et al and 2017 | 1 | 1 |
| **3** | Chawla, Rachit. et al and 2010 | 1 | 1 |
| **4** | Desalu, O. O. et al and 2016 | 1 | 1 |
| **5** | Li, Huiqin. et al and 2016 | 1 | 1 |
| **6** | Loh, Jia Fui. et al and 2018 | 1 | 1 |
| **7** | Naskar, Subrata. et al and 2017 | 1 | 1 |
| **8** | Shankar, Abhishek. et al and 2016 | 1 | 1 |
| **9** | Zainuddin, Norafiza. et al and 2018 | 0 | 1 |

CALCULATIONS FOR DEGREE OF AGREEMENT USING STATA 13

kap Reviewer1Response Reviewer2Response

Expected

Agreement Agreement Kappa Std. Err. Z Prob>Z

-----------------------------------------------------------------

77.78% 80.25% -0.1250 0.3333 -0.38 0.6462

. mcc Reviewer1Response Reviewer2Response

| Controls |

Cases | Exposed Unexposed | Total

-----------------+------------------------+------------

Exposed | 7 1 | 8

Unexposed | 1 0 | 1

-----------------+------------------------+------------

Total | 8 1 | 9

McNemar's chi2(1) = 0.00 Prob > chi2 = 1.0000

Exact McNemar significance probability = 1.0000

Proportion with factor

Cases .8888889

Controls .8888889 [95% Conf. Interval]

--------- --------------------

difference 0 -.4190897 .4190897

ratio 1 .7071758 1.414075

rel. diff. 0 -2.771808 2.771808

odds ratio 1 .0127394 78.49684 (exact)

**RESULTS INTERPRETATION**

Analysis of the results of full article screening show that there was 77.78% agreement versus 80.25% expected by chance which constitutes a considerably poor agreement between screeners (Kappa statistic = - 0. 13 and p-value >0.05). However, the McNemar's chi-square statistic suggests that there is not a statistically significant difference in the proportions of yes/no answers by reviewer with p-value >0.05.
